# Temporal reprogramming of calcium signalling via crosstalk of gonadotrophin receptors that associate as functionally asymmetric heteromers

**DOI:** 10.1038/s41598-018-20722-5

**Published:** 2018-02-02

**Authors:** K. C. Jonas, S. Chen, M. Virta, J. Mora, S. Franks, I. Huhtaniemi, A. C. Hanyaloglu

**Affiliations:** 1 Centre for Medical and Biomedical Education, St George’s, University of London, London, UK; 2 Institute of Reproductive and Developmental Biology, Dept. Surgery and Cancer, Imperial College London, London, UK; 3 Roche Pharma Research and Early Development, Roche Innovation Center Zürich, Zürich, Switzerland

## Abstract

Signal crosstalk between distinct G protein-coupled receptors (GPCRs) is one mechanism that underlies pleiotropic signalling. Such crosstalk is also pertinent for GPCRs activated by gonadotrophic hormones; follicle-stimulating hormone (FSH) and luteinising hormone (LH), with specific relevance to female reproduction. Here, we demonstrate that gonadotrophin receptor crosstalk alters LH-induced Gαq/11-calcium profiles. LH-induced calcium signals in both heterologous and primary human granulosa cells were prolonged by FSHR coexpression via influx of extracellular calcium in a receptor specific manner. LHR/FSHR crosstalk involves Gαq/11 activation as a Gαq/11 inhibitor abolished calcium responses. Interestingly, the enhanced LH-mediated calcium signalling induced by FSHR co-expression was dependent on intracellular calcium store release and involved Gβγ. Biophysical analysis of receptor and Gαq interactions indicated that ligand-dependent association between LHR and Gαq was rearranged in the presence of FSHR, enabling FSHR to closely associate with Gαq following LHR activation. This suggests that crosstalk may occur via close associations as heteromers. Super-resolution imaging revealed that LHR and FSHR formed constitutive heteromers at the plasma membrane. Intriguingly, the ratio of LHR:FSHR in heterotetramers was specifically altered following LH treatment. We propose that functionally significant FSHR/LHR crosstalk reprograms LH-mediated calcium signalling at the interface of receptor-G protein via formation of asymmetric complexes.

## Introduction

How individual cells integrate and decode multiple signals from an array of distinct receptors is a fundamental question in cell biology. This is especially relevant for the superfamily of G protein-coupled receptors (GPCRs) that represent the largest family of signalling receptors with abundant and widespread expression in all physiological systems^1,2^. Anatomical ‘atlases’ of GPCR expression in diverse tissues suggest more than 100 different GPCRs are coexpressed in any one cell type, and that systems have distinct GPCR expression signatures^3,4^. Furthermore, one mechanism contributing to this pleiotropy in cell signalling is crosstalk of GPCR signalling, and that such crosstalk can occur via GPCR heteromerisation. The latter is defined as a complex of at least two different receptor protomers, with distinct biochemical properties from its individual components or homomers^5^. These GPCR heteromeric complexes can alter receptor function from monomeric or homo-di/oligomeric complexes, at multiple levels from ligand binding, G protein activation, receptor trafficking, and may possibly play a role in biased signalling^5–9^.

Crosstalk is highly pertinent for the GPCRs that play key roles in female reproduction and pregnancy. Upon ovarian follicular maturation, the follicle-stimulating hormone receptor (FSHR) and luteinising hormone receptor (LHR) are expressed in granulosa cells sequentially, starting with FSHR expression in early follicles, progressing to a stage of FSHR and LHR coexpression in preovulatory and luteinising follicles, and subsequently to LHR being primarily expressed in luteinised follicles^10–12^. These hormones and their receptors play a central role in the regulation of sex steroid production, development of ovarian follicles, ovulation, and maintenance of corpus luteum and corpus luteum of pregnancy^11,13^. Thus, when coexpressed, heteromerisation of LHR FSHR is possible, but very little is known about their potential functional crosstalk.

The primary G protein signalling pathway that the gonadotrophin receptors are coupled to is Gαs, which activates adenylyl cyclase and increases intracellular levels of cAMP. However, for the LHR, under conditions of high hormone concentrations and high receptor expression levels, this receptor also couples to Gαq/11 to activate phospholipase C which increases levels of diacyl glycerol and inositol phosphates that trigger release of calcium (Ca^2+^) from intracellular stores; physiological relevant conditions that occur in the mural granulosa cells of the ovulatory follicle and the LH surge leading to ovulation^12,14,15^. We and others have demonstrated that LHR and FSHR are able to form constitutive homomers and heteromers^16–21^. Furthermore, we have demonstrated that the molecular composition of LHR protomers in lower order oligomers may modulate G protein activity^19^, highlighting the potential significance of gonadotrophin receptor organization in regulating signal crosstalk. LHR/FSHR crosstalk has been shown to negatively cross-modulate Gαs-cAMP signalling^20^. This inhibition of the primary G protein-signalling pathway by each receptor raises an interesting question in how such crosstalk may impact Gαq/11 signalling that is also key to follicular function.

To understand how GPCR crosstalk may impact gonadotrophin function, we have assessed whether LH-mediated Gαq/11- Ca^2+^ signalling is modulated by FSHR coexpression and dissected the underlying molecular mechanisms. We demonstrate that LH-induced Ca^2+^ signal profiles in heterologous and primary human granulosa cells are prolonged by FSHR via influx of extracellular Ca^2+^. LHR/FSHR crosstalk requires a Gαq/11 and Gβγ-dependent mechanism. Employing biophysical and super-resolution technologies, we demonstrate that LHR and FSHR form functional asymmetric complexes with Gαq, and that these receptors form distinct ligand-dependent heterotetrameric profiles.

## Materials and Methods

### Materials

Recombinant LH and FSH was from the National Peptides and Hormones Program (c/o A. F. Parlow, Harbor-UCLA Medical Center). CAGE 500 and 552 *N*-hydroxysuccinimide esters for antibody conjugation were from Abberior. FLAG and HA.11 primary antibodies were purchased from Sigma-Aldrich (UK), and Cambridge BioScience, respectively. Coelenterazine h was purchased from Promega (UK), for BRET assays, and for Ca^2+^ imaging, Fluo-4AM direct was obtained from Invitrogen. Pharmacological inhibitors nifedipine and 2-aminoethyl diphenylborinate (2-APB) was purchased from Sigma Aldrich (UK), thapsigargin from Abcam (UK) and gallein from Calbiochem (UK). The Gαq/11 inhibitor FR900359 (UBO-QIC) was purchased from the Institute of Pharmaceutical Biology, University of Bonn.

### Plasmids

N-terminally FLAG-tagged human (h) LHR, and HA-tagged hFSHR were generated as described previously^22^. FLAG-tagged β2-adrenergic receptor was kindly provided by Prof. M. von Zastrow (University of California, San Francisco). *Renilla* luciferase 8 (Rluc8) and mVenus was provided by S. Gambhir (Stanford Univ. School of Medicine, Palo Alto, CA) and A. Miyawaki (RIKEN Brain Science Institute, Japan), respectively. To generate C-terminally mVenus-tagged or Rluc8 FSHR and LHR, PCR was conducted to remove the stop codon, and sub-cloned into pcDNA3.1 plasmid containing Rluc8 or mVenus. Plasmid integrity was confirmed via sequencing. Plasmid DNA encoding Gα_q_ and Gα_s_ mVenus-tagged constructs and the untagged Gβ_1_ and Gγ_2_ subunits provided courtesy of J. Javitch (Columbia Univ. School of Medicine, New York)^23^.

### Cell Culture and Transfections

HEK 293 cells were maintained and cultured as previously described^19^. Cell lines stably expressing FLAG-hLHR or HA-FSHR were utilised for all functional studies. Stable cell lines were generated via Lipofectamine 2000® (Invitrogen)-based transfection of either FLAG-LHR or HA-FSHR plasmid DNAs, followed by G418 selection and assessment of cell surface LHR and FSHR expression levels by flow cytometry (FACSCalibur, BD Biosciences). Transient transfections were also conducted using Lipofectamine 2000® (as per manufacturer’s protocol) and experiments performed 48 h after transfection.

Human granulosa-lutein cells were harvested from follicular aspirates obtained from women undergoing controlled ovarian hyperstimulation for IVF-embryo transfer under the approved study by the National Health Service National Research Ethics-Hammersmith and Queen Charlotte’s and Chelsea Research Ethics Committee (08/H0707/152). Written informed consent was obtained from all participants in accordance with the guidelines in The Declaration of Helsinki 2000. Subjects with idiopathic or male-factor infertility were recruited from the Infertility Clinic at Hammersmith Hospital, Imperial College London NHS Trust. Cells were isolated via Percoll density gradient as previously described^24^.

### Bioluminescence resonance energy transfer (BRET)

BRET^1^ was utilised to determine ligand-mediated receptor-Gα protein interactions, as described previously^19^. Briefly, cells were rinsed and harvested into PBS, and transferred into 96-well plates at a density of ∼200,000 cells/well. Following addition of Coelenterazine h substrate, the baseline BRET ratio (475 nm/535 nm) was recorded for 1 min, before addition of ligand and further recording for an additional 1 min. To calculate BRET signals, the values at 535 nm were divided by that omitted at 475 nm. For assessing constitutive receptor-G protein or receptor-receptor associations, net BRET values were calculated by subtracting the basal BRET ratio of receptor-tagged Rluc8 alone from all readings. The effect of ligand addition were assessed by subtracting basal net BRET readings from ligand-stimulated values, followed by subtracting any changes observed with control, PBS-treated wells. Constitutive receptor-G protein association readings were carried out in duplicate and ligand-induced BRET readings in triplicate.

### Signalling Assays

To assess changes in intracellular Ca^2+^ levels, Fluo4-AM Direct (Invitrogen) labelling was conducted, following the manufacturer’s protocol. Cells were incubated with Fluo4-AM Direct at 37 °C, for 30 min, followed by incubation at room temperature for an additional 30 min. Following incubation, agonist-dependent Ca^2+^ mobilization was assessed using a TCS-SP5 confocal microscope (Leica), with × 20 dry objective, capturing 1 frame per 1.2 s. Baseline Ca2+ was assessed for ~1 min prior to agonist treatment, and the effects of agonist assessed for a further 10 min. The inbuilt Leica LASAF analytical software was used to analyse the resulting time-lapse series. Intracellular cAMP concentrations were assessed by EIA as previously described^25^, with determined cAMP concentrations controlled for any variation in protein levels.

### PD-PALM

PD-PALM studies were conducted as previously described^19^. Briefly, FLAG and HA.11 primary antibodies were labelled with CAGE 500 and 552 photoswitchable dyes, respectively, following manufacturer’s protocol (Abberior). The degree of labelling was determined to be 1.0 ± 0.2 dye molecules per antibody for FLAG-CAGE 500 and 1.3 ± 0.1 dye molecules per antibody for HA.11-CAGE 552. Cells were plated onto 8-well 1.5 borosilicate coverglass chamber slides (Labtek) and incubated with HA.11-CAGE 552/FLAG-CAGE 500 antibodies for direct labelling of FSHR and LHR, respectively, for 30 min at 37 °C prior to ligand treatment. Subsequently, cells were washed with PBS and fixed for 30 min in 4% paraformaldehyde/0.2% glutaraldehyde. Cells were washed in PBS, and maintained in PBS for imaging. Images were acquired using an inverted Axiovert 200 manual wide-field fluorescent microscope (Zeiss, Germany) fitted with a commercial TIRF condenser (TILL Photonics GmbH, Germany), and a ×100 oil immersion, 1.45 numerical aperture objective. Image acquisition was as previously described^19^. Bright field images of each series were also acquired and grid images used post-acquisition to align CAGE 500 and 552 channels, using Fiji software.

### Localization Analysis

Fluorescent intensity images of cropped non-overlapping 7 × 7 μm areas, within cell-cell boundaries, from 491- and 561-nm channels were analysed for localized receptors by QuickPALM Fiji plugin as previously described^19^. Data tables containing the x-y coordinates of localized receptors in 491- and 561-nm channels generated.

The number of associated receptor protomers derived from the *x-y* particle localization data tables were analysed using a custom Java application (PD-Interpreter), as previously described^19,26^, and coordinates from 491 and 561 nm fields plotted as an image. To determine the percentage and nature of FSH/LHR heteromerisation, a second order Getis Franklin neighbourhood analysis was conducted, using a search radius of 50 nm^19^. Outputted data were represented as a co-localization plot with differential colours distinguishing 491 and 561 channels, and heat maps generated to depict and represent the different numbers of associated receptor protomers observed.

### Flow Cytometry

To quantify the cell surface expression levels of epitope-tagged receptors in stable cell lines and transient transfections, flow cytometry was carried out using a FACSCalibur flow cytometer (BD Biosciences), as previously described^25^. The fluorescence intensity of 10,000 cells was collected per sample, and technical replicates performed in triplicate.

### Statistical analysis

For analysis of Ca^2+^ data, baseline fluorescence was subtracted and area under the curve assessed for 20 cell areas for n = 3–5. To prevent the introduction of possible bias into the analysis, the presence of an LH-induced calcium response was visually confirmed (via change in fluorescence), and subsequent cell areas randomly selected and individual cell responses analysed. Area under the curve measurements were compared by One-way ANOVA with Dunnett’s post-hoc multiple comparison test. For cAMP analysis, ligand-induced cAMP levels were expressed as fold change over basal levels, and all conditions carried out in triplicate and compared by One-way ANOVA with Dunnett’s post-hoc multiple comparison test comparing different levels of FSHR transfection on LH-dependent responses. Basal and ligand-dependent PD-PALM data are represented as the mean ± S.E.M. for 10–12 cells, from 3 independent experiments. Differences in basal versus LH induced LHR/FSHR heteromerisation were assessed by one-way ANOVA with Dunnett’s multiple comparison post-hoc test, comparing different time points to basal. For assessment of LH-dependent effects on individual LHR/FSHR dimer and oligomeric complexes, data were expressed as a percentage of each form, and a two-way ANOVA with Bonferroni’s multiple comparison post hoc test conducted, analysing the effect of LH on the associated receptor forms. Receptor trimeric and tetrameric ratios *versus* the composition of oligomeric complexes were analysed using a one-way ANOVA with Dunnett’s post hoc test.

Statistical analyses were carried out using GraphPad Prism Version 7 (San Diego, CA), and statistical significance determined as *p* < 0.05. The data that support the findings of this study, and materials used, are available from the corresponding authors upon request. PD-Interpreter software is freely available to download at www.superimaging.org.

## Results

### FSHR coexpression induces sustained LH-dependent Ca^2+^ signalling via Ca^2+^channels

It is established that one of the key physiological G protein pathways activated by LH/LHR, but not by FSH/FSHR, is Gαq/11, leading to activation of PLC and increase in inositol phosphates and intracellular Ca^2+^ ^14^^,^^15^. Therefore, we assessed the impact of LHR/FSHR coexpression on LH-induced Ca^2+^ signalling in a heterologous cell system that enabled analysis of the contribution of each receptor in a controlled manner.

**Figure 1:**
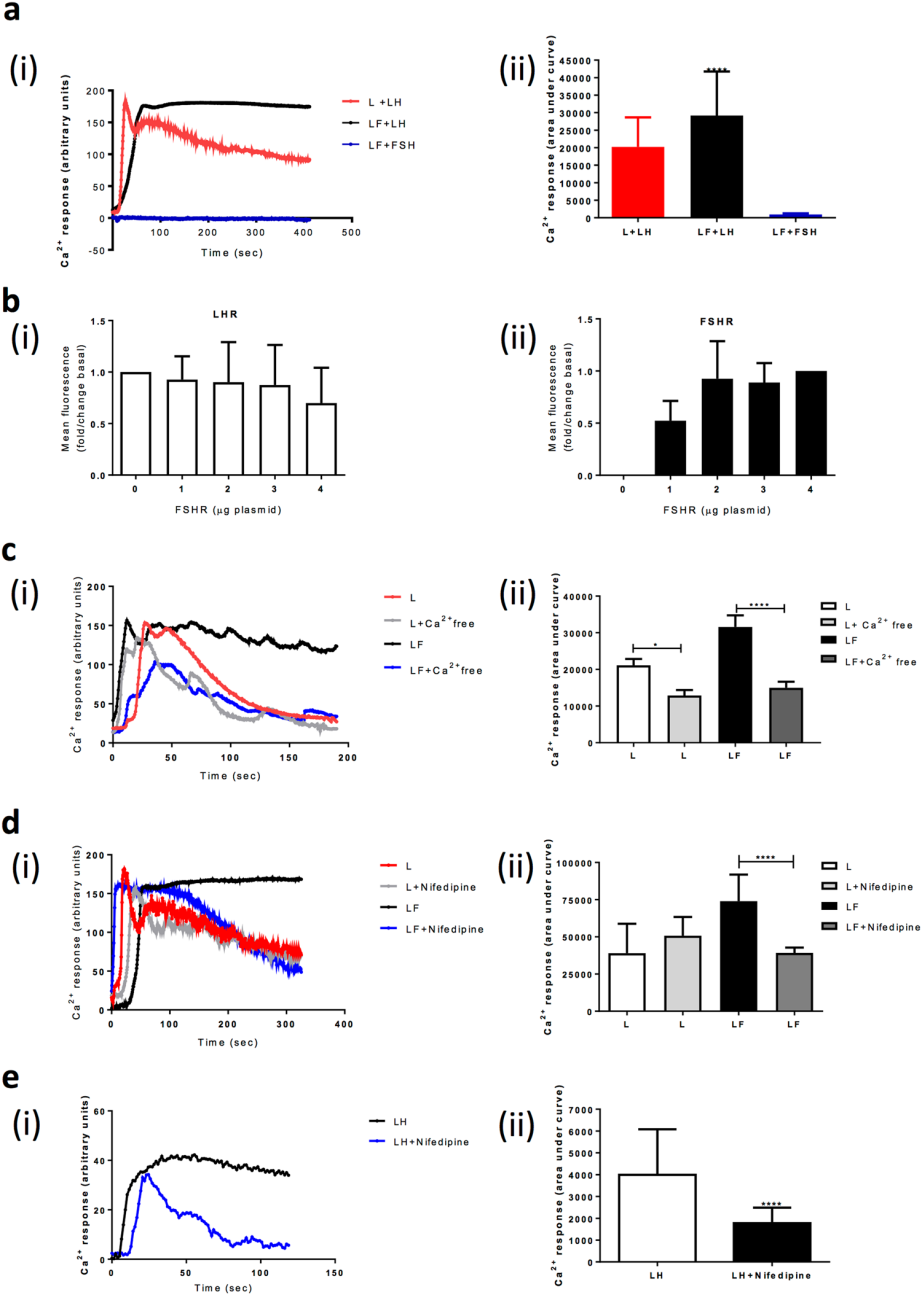
LHR/FSHR crosstalk stimulates a sustained LH-dependent Ca^2+^ release that is dependent on extracellular Ca^2+^. (**a**) (i) Cells were treated with Ca^2+^ indicator dye Fluo4-AM and imaged live via confocal microscopy. Representative Ca2+ traces showing Ca^2+^ release in cells stably expressing LHR alone (L), or co-expressing LHR/FSHR (LF) in response to LH or FSH (100 nM). Representative fluorescent trace over time with baseline fluorescence subtracted. Ligand is added at t = 0. (ii) Quantitative analysis of data as described in **a**(i). Results are expressed as area under the curve from >20 cells per condition carried out in duplicate, n = 5. Data is presented as Mean ± SEM. (**b**) Measurement of cell surface expression via flow cytometry of (i) FLAG-tagged LHR and (ii) HA-tagged FSHR, in cells stably expressing FLAG-LHR and transiently transfected with increasing amounts of HA-FSHR plasmid DNA. Mean ± SEM, n = 5 (**c**) Assessment of the sustained LH-dependent Ca^2+^ response in cells co-expressing LHR/FSHR under Ca^2+^ free PBS conditions. Cells were incubated in Fluo4-AM indicator dye in Ca^2+^ free PBS prior to live confocal imaging and stimulation with LH (100 nM). (**d**) Measurement of LH-dependent Ca^2+^ responses with and without pre-treatment with nifedipine (10 μM, 30 min). (**e**) Assessment of LH-dependent Ca^2+^ responses in primary human granulosa-lutein cells co-expressing LHR/FSHR treated as in (**d**). Representative fluorescent traces over time with baseline fluorescence subtracted are shown in **c**(i)–**e**(i). For **a**(ii) and **c**(ii)–**e**(ii), results are expressed as area under the curve from n = 3–5 experiments, *p < 0.05, ****p < 0.0001.

FLAG-tagged human LHR stably expressed in HEK 293 cells were treated with a Ca^2+^ indicator dye, Fluo4-AM, and imaged live via confocal microscopy. LHR induces Gαq/11-Ca^2+^ signalling only under high receptor and high hormone concentrations^15,19^. This cell line expressed at the plasma membrane between 4000–10, 000 receptors/cell (as assessed by PD-PALM), which is consistent with physiological levels reported for the ovary^27^. Stimulation of LHR-expressing cells with 100 nM LH induced a rapid increase in intracellular Ca^2+^ levels that declines within 20 sec of the maximal fluorescent response (Fig. 1ai). Interestingly, co-expression of an HA-tagged human FSHR resulted in a sustained LH-induced Ca^2+^ signal profile (Fig. 1ai) and significantly increased the area under the Ca^2+^ response curve (Fig. 1aii). This increase was specific to LHR activation as stimulation of cells co-expressing LHR and FSHR, with FSH, failed to induce a Ca^2+^ response (Fig. 1ai). Likewise, FSH could not induce a Ca^2+^ signal in cells only expressing FSHR (Supplementary Fig. S1). Cell surface expression of both FSHR and LHR in LHR stable cells was confirmed via flow cytometry, where surface LHR levels were not significantly altered in cells co-expressing FSHR (Fig. 1b). Furthermore, co-expression of a distinct Gαs-coupled receptor, the β2-adrenergic receptor, in LHR expressing cells, did not significantly affect LH-mediated Ca^2+^ responses (Supplementary Fig. S2). In contrast to the enhanced LH-mediated Ca^2+^ profiles by FSHR, LH-dependent cAMP levels were reduced by FSHR co-expression (Supplementary Fig. S3a), consistent with prior observations^20^, and confirmed at the receptor/G protein level, as a decrease was observed in the LH-dependent associations between LHR and Gαs in the presence of FSHR, via BRET (Supplementary Fig. S3b).

To identify the mechanism for the highly-sustained LH-mediated Ca^2+^ response by FSHR co-expression, we determined whether an influx of extracellular Ca^2+^ mediates the prolonged elevated levels of intracellular Ca^2+^. Measurement of intracellular Ca^2+^ levels in cells expressing LHR and FSHR were assessed in the presence or absence of extracellular Ca^2+^. In cells expressing LHR, removal of extracellular Ca^2+^ resulted in a significant decrease in LH-dependent Ca^2+^ response, as assessed by the area under the curve (~40%). LHR/FSHR co-expressing cells exhibited a more pronounced inhibition in the LH-induced sustained response (~60%), reverting the profile from a sustained to transient response (Fig. 1c). Pre-treatment of LHR-expressing cells with nifedipine, a dihydropyridine Ca^2+^ channel blocker known to effectively inhibit endogenous Ca^2+^ channel currents in HEK 293 cells^28^, had no significant effect on LH/LHR induced Ca^2+^ maximum responses or profiles (Fig. 1d). In contrast, nifedipine pre-treatment reverted the sustained Ca^2+^ profile observed with FSHR to an ‘LHR-like only’ profile, as evidenced by a significant decrease in area under the curve without effecting the initial maximum response. This suggests that FSHR may sustain elevated LH/LHR-mediated Ca^2+^ levels via Ca^2+^ channels, in addition to release of Ca^2+^ from intracellular stores. Thus, in order to determine if intracellular calcium store release, and/or store-operated Ca^2+^ channels were involved, cells were pretreated with either; thapsigargin (to deplete intracellular Ca^2+^ stores) or 2-APB, a well characterized inhibitor of store-operated, or voltage-independent, Ca^2+^ channels in an IP_3_ receptor-independent manner^29–32^. Thapsigargin dramatically inhibited LH-induced increases in intracellular Ca^2+^ in cells expressing either LHR, or both LHR and FSHR (Supplementary Fig. S4a). Interestingly, 2-APB inhibited only the sustained Ca^2+^ signal induced by FSHR (Supplementary Fig. S4b), in a similar manner as pre-treatment with nifedipine. Nifedipine has reported to inhibit distinct types of Ca^2+^ channels, including voltage-independent and store-operated channels^33–37^. To address whether this profile of LH-induced Ca^2+^ signalling was evident in a physiological relevant cell type, Ca^2+^ responses were measured in primary human granulosa-lutein cells known to express both receptors (Fig. 1e). LH-dependent Ca^2+^ responses were sustained and inhibited by pre-treatment with nifedipine, suggesting LH/LHR-mediated Ca^2+^ signalling in these cultures exhibit similar properties to that observed in LHR/FSHR expressing HEK 293 cells.

### Sustained LH-dependent Ca^2+^ signalling, via FSHR, is Gαq/11- and Gβγ-dependent

**Figure 2:**
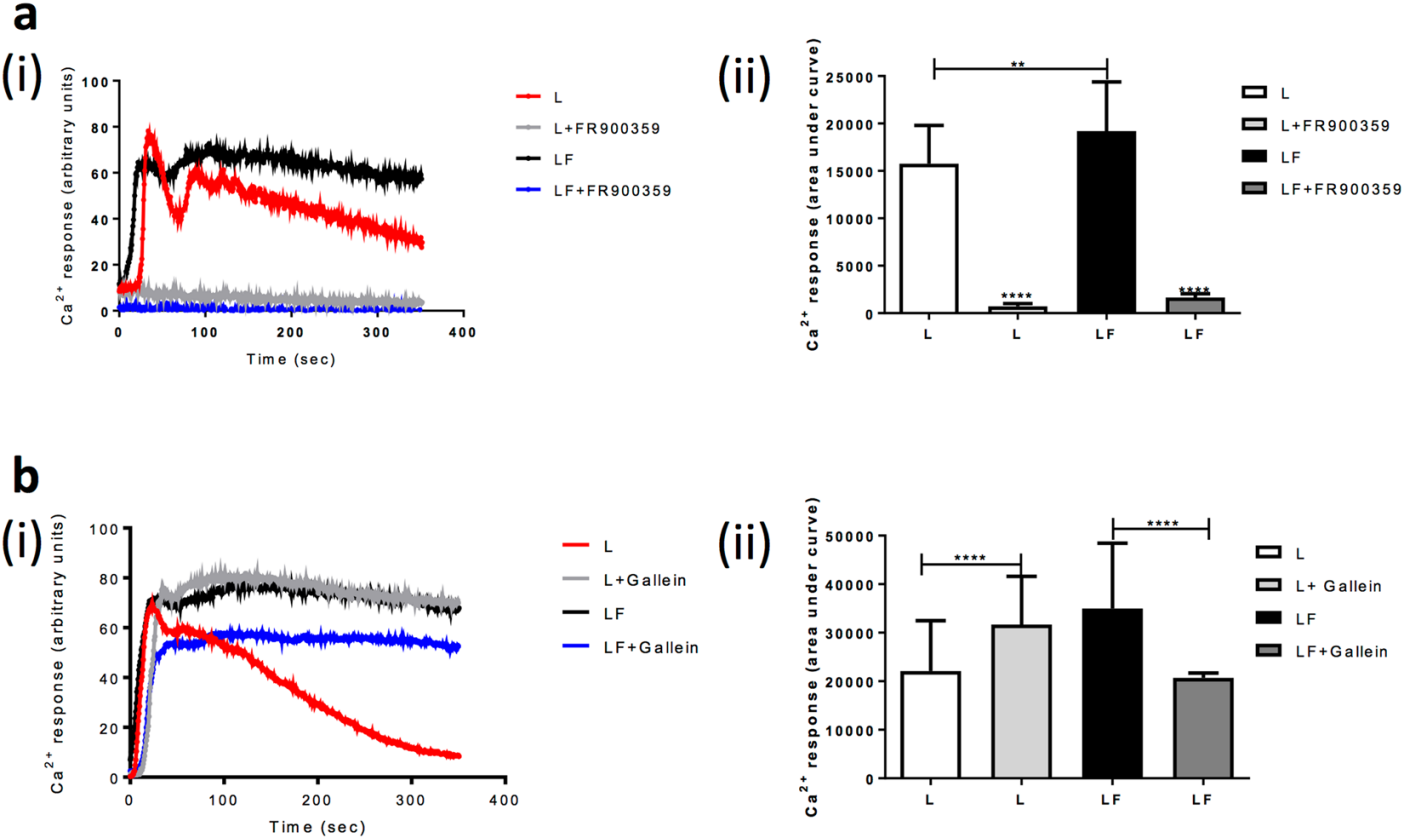
Sustained LHR/FSHR Ca2 + release is Gαq/11 and Gβγ-dependent. (**a**,**b**) HEK 293 cells stably expressing LHR were pre-treated with FR900359, a Gαq/11 inhibitor (**a**) (1 μM, 2 h) or a Gβγ inhibitor gallein (**b**) (1 h) and intracellular Ca^2+^ levels in response to LH (100 nM) were measured. Representative fluorescent traces over time with baseline fluorescence subtracted are shown in **a**(i) and **b**(i). For **a**(ii) and **b**(ii) results are expressed as area under the curve from n = 3–5 experiments. **p < 0.01, ****p < 0.0001.

GPCR crosstalk has been shown to alter the G protein-complement^5^. For LHR, the ligand-dependent increase in Ca^2+^ occurs via activation of Gαq/11. Thus, to determine if the alteration in LHR-mediated Ca^2+^ signalling, by FSHR co-expression, involves Gαq/11 function, a potent and established inhibitor, FR900359^38^, was used. Pre-treatment of cells stably expressing LHR with FR900359, with or without FSHR co-expression, dramatically inhibited both the acute and sustained LH-induced Ca^2+^ responses seen in LHR/FSHR expressing cells (Fig. 2a), thus demonstrating the critical dependence on Gαq/11. However, to understand how the sustained Ca^2+^-response was mediated we assessed the role of Gβγ, which acts as an effector for distinct pathways including activation of PLCβ and modulation of Ca^2+^ channels^39,40^. Treatment with the Gβγ inhibitor gallein induced unexpected opposing effects on the LH-mediated Ca^2+^ response, producing a sustained response in cells expressing LHR alone, yet significantly inhibiting the Ca^2+^ response in cells co-expressing LHR and FSHR (Fig. 2b). The partial inhibition by gallein may be due to the opposing increases in Ca^2+^ levels induced by LHR only complexes. Overall, this suggests that in the presence of the FSHR, LH-induced activation of sustained Ca^2+^ responses may also require Gβγ activation.

### FSHR induces a rearrangement in ligand-induced LHR-Gαq associations

**Figure 3:**
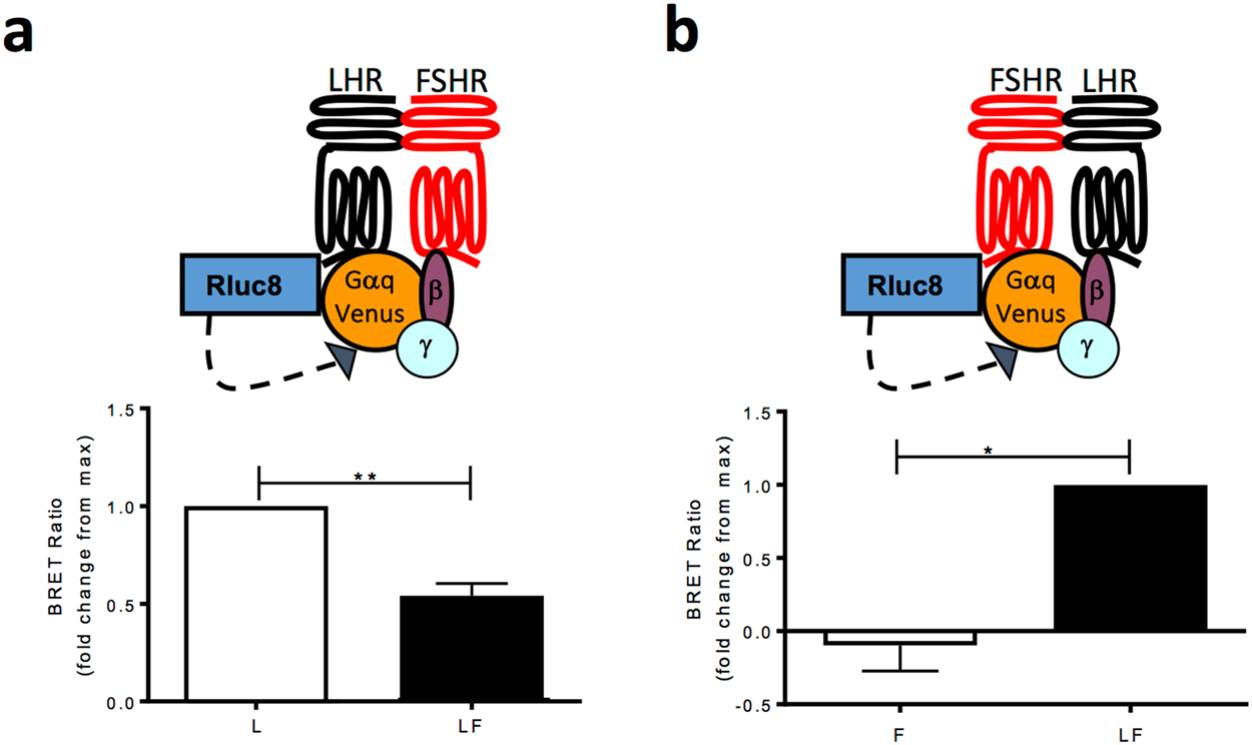
LH-dependent association of LHR with Gαq is rearranged in the presence of FSHR. Schematic representation and BRET analysis of LH-dependent association of Gαq-Venus with either LHR-Rluc8 **(a)** or FSHR-Rluc8 **(b)**, expressed in HEK 293 cells stably expressing FSHR or LHR, respectively or in HEK 293 cells. L, LHR-Rluc8 transiently expressed in HEK 293 cells; F, FSHR-Rluc8 transiently expressed in HEK 293 cells; LF, either LHR-Rluc8 or FSHR-Rluc8 in cells stably expressing FSHR or LHR, respectively. Basal BRET was obtained for 1 min prior to LH (100 nm) addition for 1 min and treatments carried out in triplicate. Data is represented as mean ± SEM from n = 3. *p < 0.05, **p < 0.01.

In addition to both transient and sustained (LHR/FSHR-mediated) Ca^2+^ responses being Gαq/11-dependent, we next asked if there were alterations at the level of G protein coupling to LHR, in the presence of FSHR, that underlie this positive modulation of LH/LHR signalling by FSHR. To do this we measured ligand-dependent receptor-Gαq associations via BRET^19^. Both LHR and FSHR were C-terminally tagged with the donor Rluc8 and expressed with Venus tagged Gαq. The BRET-tagged constructs were expressed in HEK 293 cells stably expressing either LHR or FSHR (Fig. 3) to determine whether activation is altered in the presence of the other receptor. As we have previously shown^19^, 100 nM LH induces an increase in the BRET signal between LHR-Rluc8 and Gαq-Venus (Fig. 3a). Surprisingly, there was a significant decrease in the LH-induced BRET in the presence of the FSHR (Fig. 3a). This decrease may be due to a reorganization of LHR/Gαq complexes with FSHR, as a ligand-induced BRET signal was observed between FSHR-Rluc8 and Gαq-Venus in the presence of LHR, and only following LH activation (Fig. 3b). Overall, this data suggests a rearrangement in proximity between ligand-activated LHR and Gαq via close associations with FSHR.

### Super-resolution imaging of LHR and FSHR reveals preformed heteromers modulated by ligand

The BRET data suggest that LH-dependent sustained Ca^2+^ signalling and altered G protein coupling may occur via formation of LHR/FSHR heteromers. These receptors are known to interact via BRET and fluorescence correlation spectroscopy approaches^20,21^ and confirmed in this study also via BRET (Supplementary Fig. S5). However, we have recently reported the technique of two colour PD-PALM to study LHR homomers that enables quantitation of individual receptors in distinct complexes with functional asymmetry^19^. Due to the degree of precision and resolution (<10 nm) of this technique, we are able to identify and quantify dimers and low order oligomers, i.e. trimers and tetramers, that have different protomer compositions at the cell surface^19,26^. As LH-mediated Ca^2+^ responses and Gαq-associations are altered by FSHR, we employed PD-PALM for its ability to detect rearrangements in heteromeric makeup following ligand-dependent activation of LHR.

**Figure 4:**
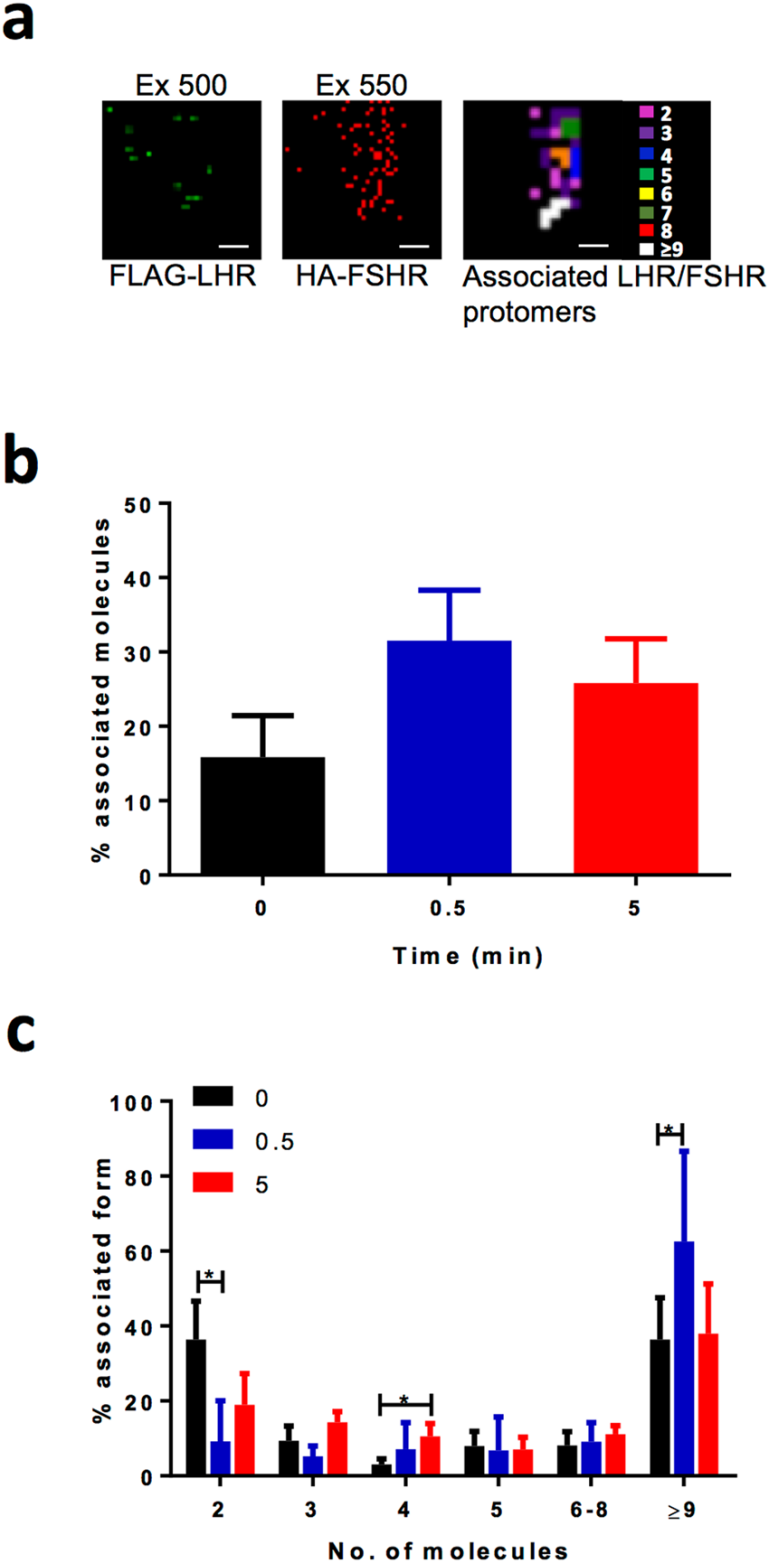
PD-PALM analysis of basal and LH-dependent cell surface LHR/FSHR heteromers. (**a**) Representative PD-PALM images and associated molecule heat maps from cells stably expressing FLAG-LHR and transiently expressing HA-FSHR. Cells were labelled live with CAGE500-conjugated FLAG and CAGE552-conjugated HA antibodies for 30 min at 37 °C prior to LH stimulation (100 nM at 0, 0.5 or 5 min). (**b**) Comparison of the percentage of total associated heteromeric molecules in cell lines stably expressing FLAG-LHR with FSHR via PD-PALM in the presence and absence of 100 nM LH treatment for 0, 0.5, or 5 min. (**c**) Analysis of the individual LHR/FSHR hetero-dimeric and hetero-oligomeric from (**b**). Each data point represents mean ± SEM from 8–12 cells, n = 3. *p < 0.05.

To analyse the types of complexes that LHR and FSHR associate at the plasma membrane, we carried out dual colour PD-PALM in cells co-expressing FLAG-LHR and HA-FSHR using directly conjugated CAGE dyes 500 and 552 that we have previously characterized^19^ (Fig. 4a). We first analysed the plasma membrane organization of LHR and FSHR heteromers, in the absence of ligand, and quantified that 17% formed heteromeric complexes with the remaining populations either monomers or homomers (Fig. 4b). Stimulation of cells with LH did not significantly alter the overall levels of LHR-FSHR associations (Fig. 4b). Quantitation of individual receptor complexes, either before or after LH treatment, revealed a significant decrease in the number of heterodimers following 30 seconds of LH treatment, possibly due to an increase in the percentage of higher order complexes or clusters (>9 receptors/complex). This increase was not observed following 5 min of ligand stimulation. Interestingly, there was a significant increase in heterotetramers after 5 min of LH treatment (Fig. 4c).

**Figure 5:**
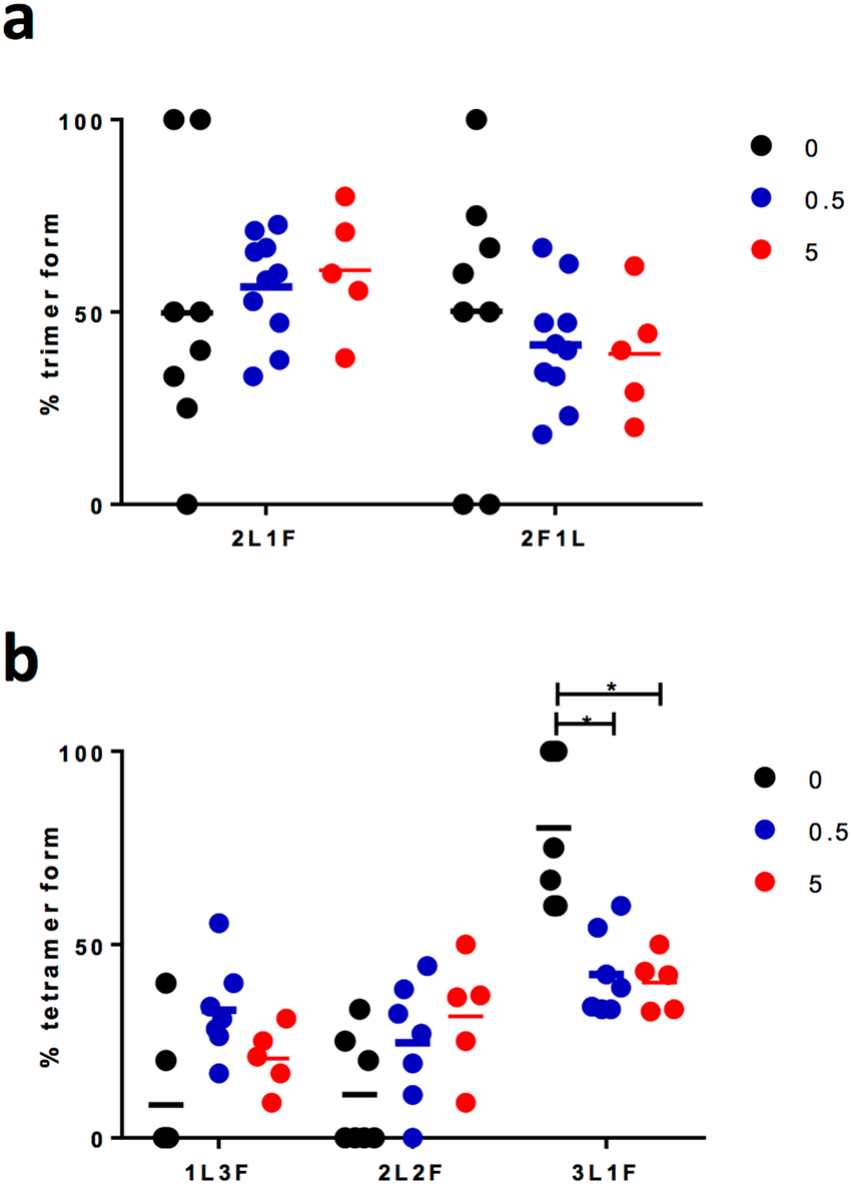
LHR/FSHR tetramers, but not trimers, exhibit LH-dependent re-arrangement. Analysis of basal and LH-treated PD-PALM datasets, as decribed in Fig. 4, to determine the ratiometric composition of LHR/FSHR trimers (**a**) and tetramers (**b**). Each data point represents an individual cell, n = 5–10 cells across 3 independent experiments. *p < 0.05.

We have previously shown that gonadotrophin receptor complexes with >6 receptors are dependent on receptor density, while the lower order complexes (2–5 receptors) are not^19^. Therefore, we further analysed the LHR:FSHR composition of both trimers and tetramers. Quantification of the ratiometric composition of heterotrimers, before and after LH treatment, revealed that complexes were comprised of equivalent levels of 2 LHRs:1 FSHR and 2 FSHRs:1 LHR. However, the relative amounts of each form of trimer were not significantly altered by LH treatment (Fig. 5a). Interestingly, analysis of heterotetramers revealed that the predominant form prior to ligand treatment is 3LHRs:1FSHR, observed in ~80% of heterotetramers (Fig. 5b). Following stimulation of cells with LH at either time point, there was a significant decrease in the levels of this heterotetramer, resulting in an equal distribution of the three distinct heterotetrameric forms (Fig. 5b). Overall, this data demonstrates that LHR and FSHR form distinct receptor complexes and that only a subset of these oligomers is altered at the protomer level by LH.

## Discussion

GPCRs exhibit remarkable signal pleiotropy given the limited set of heterotrimeric G protein signalling pathways they couple to. One key mechanism that has emerged underlying this signal diversity is receptor crosstalk, with an increasing number of reports identifying that this occurs at the receptor level via GPCR heteromerisation^5–9^. Crosstalk is highly pertinent for the GPCRs playing multiple key roles in both reproduction and pregnancy, which includes the gonadotrophin hormone receptors LHR and FSHR. Here we describe a mechanism for GPCR crosstalk resulting in positive modulation of Ca^2+^ signalling and formation of functional asymmetry at the interface of receptor-receptor and receptor-G protein organization.

In this study, we demonstrate that the unliganded FSHR can dramatically alter the Gαq/11-dependent Ca^2+^ signalling profile of the ligand-activated LHR. That an unliganded FSHR can alter LH/LHR Ca^2+^ signalling may suggest that this crosstalk is mediated by direct associations of FSHR with LHR that could impose a conformational change in LHR, effectively acting as an allosteric modulator. Additionally, as the glycoprotein hormone receptors exhibit strong negative cooperativity in binding^41^, LHR/FSHR crosstalk may favour LH binding, given that LHR, when expressed with FSHR, can increase dissociation of FSH from its receptor^20^. LHR and FSHR have been shown to exhibit cross-inhibitory effects on Gαs-cAMP signalling^20^, and confirmed in this study. Thus, in combination with our data demonstrating altered LH-dependent Ca^2+^ signalling, suggests that FSHR may positively modulate LH/LHR signalling, perhaps specifically to Gαq/11-βγ-Ca^2+^ signalling, while reciprocally LHR may negatively regulate FSHR-mediated cAMP signalling^20^.

In the context of granulosa cells that co-express these receptors, one could speculate that gonadotrophin receptor crosstalk alters LH signalling during the switch from FSH/FSHR to LH/LHR dominance at a functional level, towards defined pathways required for ovulation and subsequent granulosa cell luteinisation. That we observe a similar profile of LH-mediated Ca^2+^ signalling in primary human granulosa-lutein cells supports these findings. Attempts to manipulate LHR/FSHR levels in these primary human cultures to directly demonstrate a requirement for both receptors in the LH-induced Ca^2+^ profile, however, have been unsuccessful.

The underlying mechanism of sustained Ca^2+^ signalling by LHR/FSHR crosstalk is via Gαq/11, as a specific Gαq/11 inhibitor inhibited both the transient and sustained Ca^2+^ responses. A release of intracellular Ca^2+^ can lead to activation of ligand-gated ion channels, receptor-operated, or store-operated Ca^2+^ channels. However, the Ca^2+^ response in cells expressing LHR alone was insensitive to the Ca^2+^ channel blockers nifedipine and 2-APB, yet partially affected by removal of extracellular Ca^2+^, suggesting that the source of Ca^2+^ flux is primarily, but not completely, from intracellular stores. In contrast, in cells expressing LHR/FSHR the sustained response is sensitive to both the removal of extracellular Ca^2+^ and these Ca^2+^ channel blockers. This suggests there may be a differential mode for LH-dependent extracellular Ca^2+^ influx due to crosstalk with FSHR. Nifedipine is conventionally described as an L-type Ca^2+^ channel antagonist, however, HEK293 cells are not thought to express L-type Ca^2+^ channels, although inhibition of endogenous Ca^2+^ influx by dihydropyridines such as nifedipine have been previously described in this cell type^28^. Interestingly, nifedipine has also been shown by several groups to inhibit store-operated, or voltage-independent, Ca^2+^ channels^33–37^, suggesting this inhibitor has broader actions across distinct channel types. As we also observed that intracellular Ca^2+^ store depletion by thapsigargin inhibits transient and sustained Ca^2+^ suggests intracellular Ca^2+^ release is a prerequisite for the influx of extracellular Ca^2+^. Although human granulosa-lutein cells have been previously shown to express functional L-type Ca^2+^ channels^42^, the broader actions of nifedipine could also suggest involvement of non-voltage dependent Ca^2+^ channels. In the presence of FSHR, sustained Ca^2+^ signalling by LH is also Gαq/11-dependent suggesting that there are perhaps alterations in LHR signalling via FSHR crosstalk downstream of Gαq/11. Interestingly, rather than downstream of Gαq/11, mechanistically the LHR/FSHR crosstalk appears to be at the G protein level because BRET demonstrated that LHR/Gαq complexes are reorganized in the presence of FSHR to favour FSHR/ Gαq couplings specifically activated by LH. Furthermore, pharmacological inhibition of Gβγ partly inhibited the Ca^2+^ response by LHR/FSHR. This inhibition was only partial as it was likely counteracted by the unexpected sustained Ca^2+^ profile observed in LHR only expressing cells, as the LHR/FSHR-mediated Ca^2+^ response would represent a combination of LHR and LHR/FSHR responses, even within an individual cell. Given the potential differential modes of extracellular Ca^2+^ influx between LHR and LHR/FSHR expressing cells, and differential sensitivity to gallein, one possibility is that Gβγ may regulate the activity of specific Ca^2+^ channels activated by LHR and LHR/FSHR in an opposing manner. This is consistent with the concept that there may be differential cellular pools of Gβγ, composed of distinct Gβγ subunit combinations, with distinct cellular signalling functions^40^. Overall, our data clearly indicates how FSHR, in the absence of agonist activation, potentially reorganizes the signal machinery to reprogram LHR activation.

The ability of FSHR to induce these alterations in LHR/Gαq/Ca^2+^ signalling suggests this may be occurring at a direct receptor-receptor level via heteromerisation. The gonadotrophin receptors have been previously demonstrated to form homomers and heteromers in heterologous cells, the latter being demonstrated via BRET and fluorescence correlation spectroscopy approaches^20,21^. These approaches have their distinct advantages and disadvantages; however, super-resolution microscopy enables direct visualization of each receptor molecule at the plasma membrane with <10 nm resolution, enabling quantitation of each receptor form. Thus, we have previously demonstrated with PD-PALM that LHR exists as monomers, dimers and various oligomeric forms^19^. Employing LHR mutants that undergo functional complementation we were able to demonstrate that oligomers with functional asymmetry could modulate LHR signalling^18,19^. In the current study, super-resolution imaging of LHR/FSHR heteromers, which are functional asymmetric for LH-mediated Gαq/Ca^2+^ signalling, revealed that basally these receptors not only formed heterodimers but also distinct hetero-oligomers. Our prior studies clearly indicate that oligomers with >6 receptors are density dependent^19^, thus, we focused on the lower order oligomeric forms of trimers and tetramers. Under conditions of LHR activation where we observe a sustained Ca^2+^ signal profile by FSHR, only the heterotetramers increased in number. This was surprising as our prior studies have demonstrated that LHR homomers detected via PD-PALM were not altered via ligand activation^19^. Another advantage of PD-PALM over other techniques to study receptor di/oligomer formation is that it enables identification of the ratio of each protomer within distinct oligomeric forms. Further analysis of LHR/FSHR heterotetramers revealed that the predominant pre-formed complex consists of tetramers with 3LHRs to 1FSHR. However, ligand activation reorganizes these specific heterotetramers so there is an equal distribution of all 3 possible heterotetramers. Although we did not directly demonstrate a specific role of heterotetramers in mediating LHR/FSHR crosstalk, it is tempting to speculate that >1 FSHR protomer within a heterotetramer is required to achieve the modulations in Ca^2+^ signalling via LHR. This is potentially consistent with previous reports suggesting the active form of FSHR is a trimer^43^. Furthermore, the known negative cooperativity of LHR^41^ would suggest only one LHR protomer in a complex (homomer or heteromer) is sufficient for binding ligand. Other reported GPCR heterotetramers are formed by dimers of two distinct GPCRs that can enable coupling to two distinct G proteins^44–46^. However, our study suggests that the protomers in a GPCR heterotetramer complex can also be asymmetric. Collectively this work illustrates that GPCRs can not only form as monomers, dimers and oligomers, but even within a single oligomeric form, e.g. tetramers, there exist multiple possible active forms that could have distinct functional properties. We and others have demonstrated that gonadotrophin receptor homomer interaction interfaces are highly complex, involving both the extracellular domain and multiple possible transmembrane domains^17,19^. We have previously proposed that such complexity enables formation of these different forms (dimer, trimer, tetramer etc), but also could enable diversity in associated forms within the context of a heteromer. The ability to identify complex-specific interacting sites for both gonadotrophin receptor homomers and heteromers remains to be determined.

To conclude, we have demonstrated that GPCRs for the gonadotrophic hormones can alter signalling in a highly asymmetric manner and that this reprogramming of Ca^2+^ signalling by LHR/FSHR is modulated at the G protein level. Furthermore, these receptors form specific hetero-tetramers in an LH-dependent manner. The distinct properties of this receptor crosstalk, possibly via highly specific heteromers, presents potential modalities to exploit this complexity and improve the specificity of current therapeutics, which is potentially applicable for many GPCRs in this superfamily.

## Electronic supplementary material

Supplementary information
