# Supplementary material for: Temporal reprogramming of calcium signalling via crosstalk of gonadotrophin receptors that associate as functionally asymmetric heteromers
Source: Sci Rep. 2018 Feb 2;8:2239. doi: 10.1038/s41598-018-20722-5 (PMC5797151; doi:10.1038/s41598-018-20722-5)
Supplement: Supplementary file 1 — Supplementary information [file 41598_2018_20722_MOESM1_ESM.pdf]

Supplementary Information

Temporal reprogramming of calcium signalling via crosstalk of gonadotrophin receptors that associate as functionally asymmetric heteromers

Jonas, K.C, Chen, S; Virta, M, Mora, J; Franks S; Huhtaniemi, I; Hanyaloglu A.C

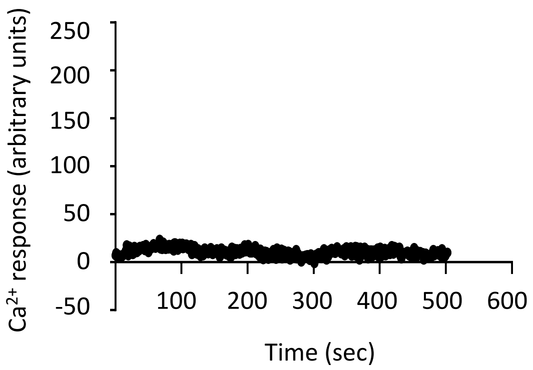

Figure S1. Activation of FSHR does not induce increases in intracellular Ca^{2+}.

Cells stably expressing HA-FSHR only were treated with Ca^{2+} indicator dye Fluo4-AM and imaged live via confocal microscopy. Cells were stimulated with FSH (100 nM). Representative fluorescent trace over time with baseline fluorescence subtracted. Ligand is added at t=0. ~20 cells were analysed per ligand treatment carried out in duplicate, n=3.

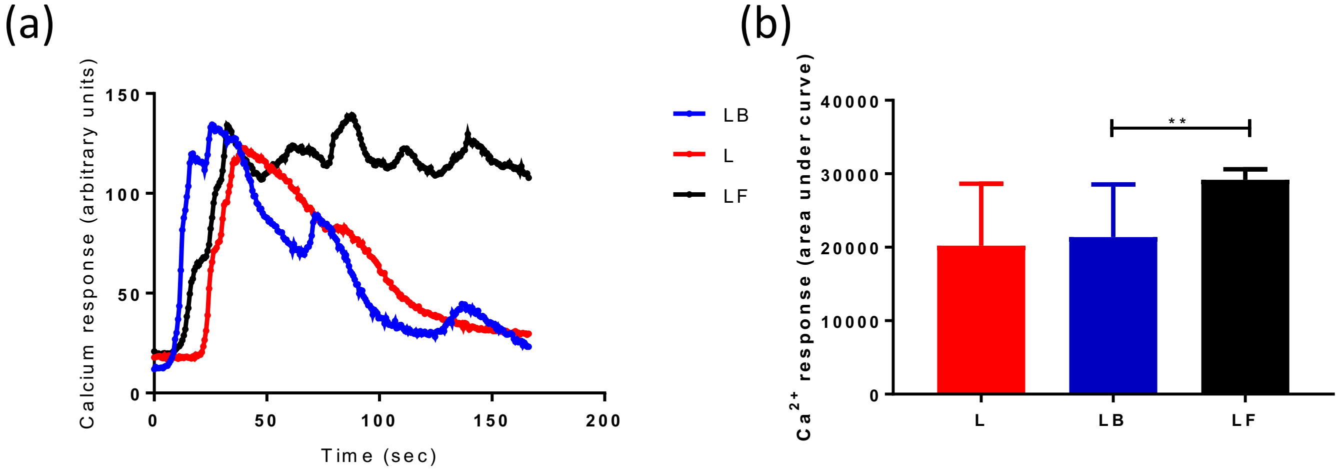

Figure S2. Coexpression of FSHR, but not the β2-adrenergic receptor, in LHR expressing cells induces a sustained LH-dependent Ca^{2+} response.

Cells stably expressing LHR only (L), or co-expressing either FSHR (LF) or the β2-adrenergic receptor (LB) were treated with Ca^{2+} indicator dye Fluo4-AM and imaged live via confocal microscopy. All cells were stimulated with LH (100 nM). (a) Representative calcium traces (b) cumulative results, expressed as area under the curve from >20 cells per condition carried out in duplicate, n=3. Data is presented as Mean ± SEM.

a

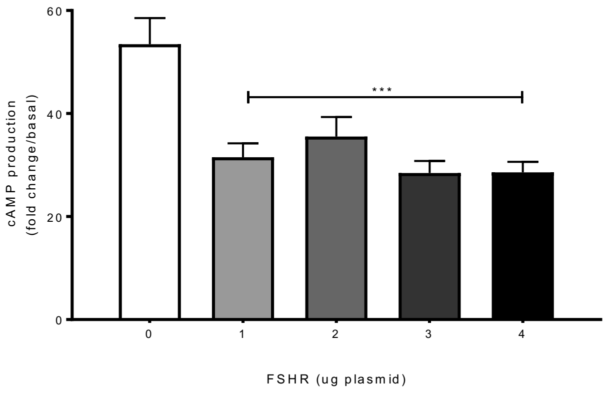

b

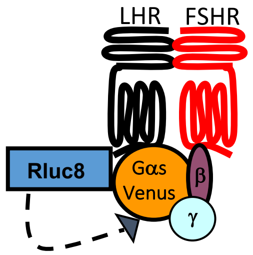

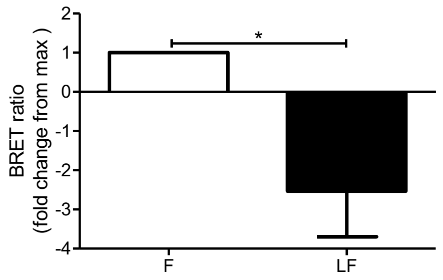

Figure S3. Crosstalk between LHR and FSHR attenuates LH/LHR-dependent cAMP production and interaction with Gαs. a. Cells stably expressing LHR were transfected with increasing amounts of FSHR plasmid as indicated in figure. Levels of intracellular cAMP were measured following pre-treatment with IBMX (0.5 mM, 15 min) followed by stimulation with LH (10 nM, 5 min). Cells were lysed in 0.1M HCL/0.1% Triton and cAMP measured via EIA and normalized to protein levels via Bradford. Results are expressed as fold change over basal. Mean + SEM, n=3. ***p<0.001, One-way ANOVA with Dunnett's post-hoc test. **b.** Schematic representation and BRET analysis of LH-dependent association of Gαs-Venus with LHR-Rluc8 expressed in HEK 293 cells or HEK 293 cells stably expressing FSHR. L, LHR-Rluc8 transiently expressed in HEK 293 cells; LF, LHR-Rluc8 in cells stably expressing FSHR. Basal BRET was obtained for 1 min prior to LH (100 nM) addition for 1 min and treatments carried out in triplicate. Data is represented as mean ± SEM from n=4. *p<0.05.

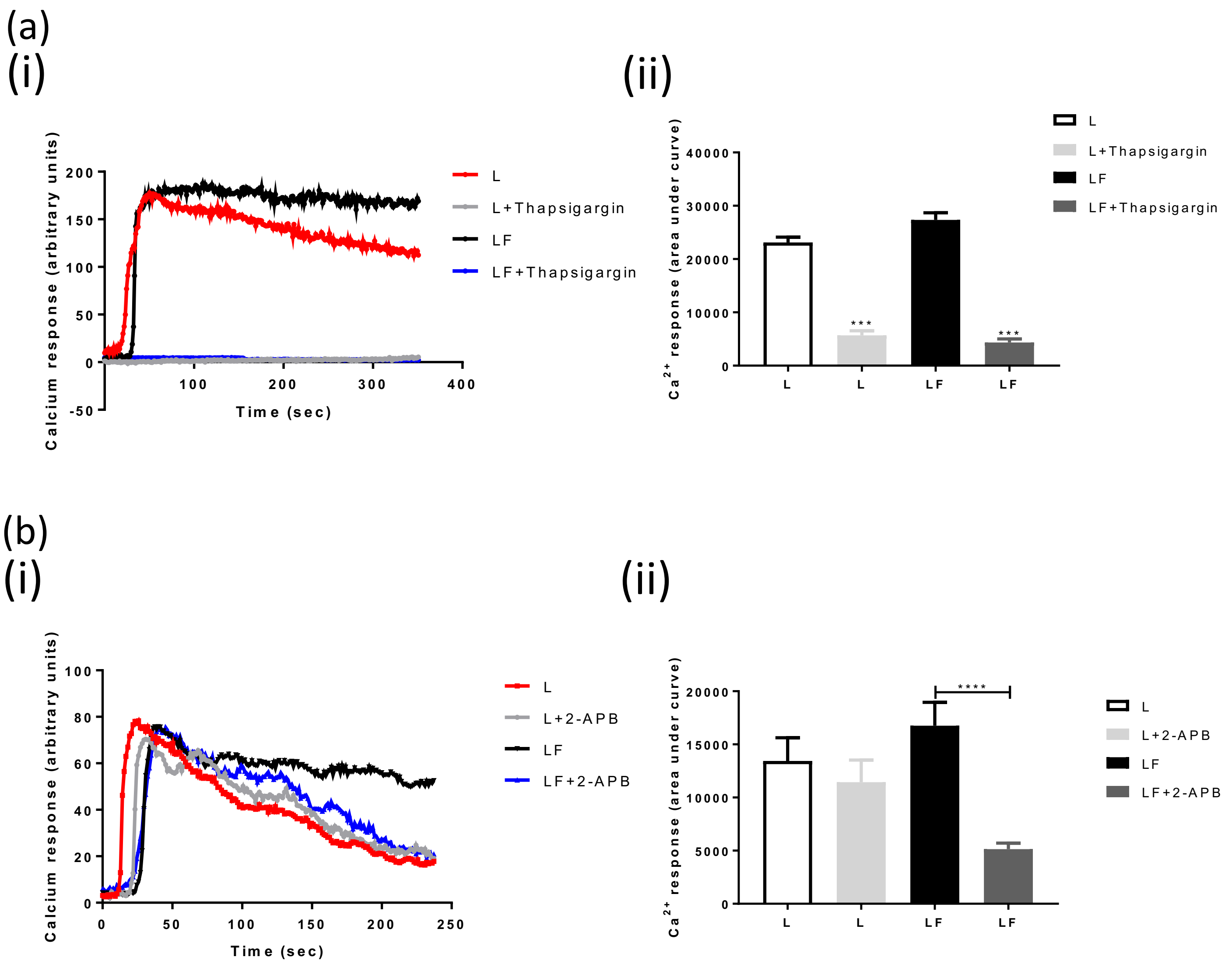

Figure S4. LH-induced Ca^{2+} response is IP3-dependent and the sustained response store-operated calcium channel mediated in LHR/FSHR co-expressing cells.

Cells stably expressing LHR only (L), or co-expressing either FSHR (LF) were treated with Ca^{2+} indicator dye Fluo4-AM and imaged live via confocal microscopy. Cells were pre-treated with **(a)** 100 μM 2-APB for 1 min or **(b)** 1 μM thapsigargin for 15 min prior to stimulation with LH (100 nM). (a(i), b(i)) Representative calcium traces (a(ii), b(ii)) cumulative results, expressed as area under the curve from >20 cells per condition carried out in duplicate, n=3. Data is presented as Mean ± SEM.

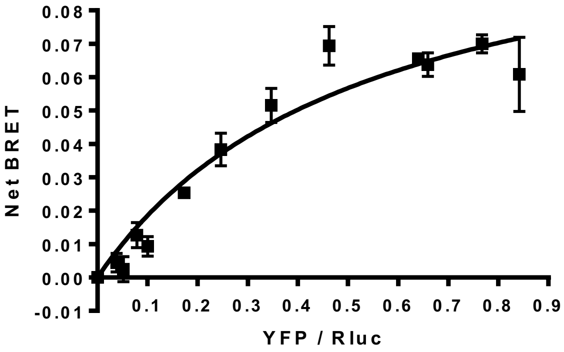

Figure S5. LHR and FSHR form heteromers. HEK 293 cells transiently expressing LHR-RLuc8 with and without increasing amounts of FSHR-Venus were assessed for their ability to associate as preformed complexes via BRET. Representative BRET saturation curve of duplicate reads over 5 independent experiments.
